# Thiol-Surface-Engineered Cellulose Nanocrystals in Favor of Copper Ion Uptake

**DOI:** 10.3390/polym15112562

**Published:** 2023-06-02

**Authors:** Trung Dang-Bao, Thi-My-Chau Nguyen, Gia-Han Hoang, Hoa-Hung Lam, Hong-Phuong Phan, Thi-Kieu-Anh Tran

**Affiliations:** 1 Faculty of Chemical Engineering, Ho Chi Minh City University of Technology (HCMUT), 268 Ly Thuong Kiet Street, District 10, Ho Chi Minh City, Vietnam; 2 Vietnam National University Ho Chi Minh City, Linh Trung Ward, Thu Duc City, Ho Chi Minh City, Vietnam

**Keywords:** cellulose nanocrystals, thiol functionalization, esterification, surface engineering, chemisorption

## Abstract

Cellulose, the most abundant natural polymer on earth, has recently gained attention for a large spectrum of applications. At a nanoscale, nanocelluloses (mainly involving cellulose nanocrystals or cellulose nanofibrils) possess many predominant features, such as highly thermal and mechanical stability, renewability, biodegradability and non-toxicity. More importantly, the surface modification of such nanocelluloses can be efficiently obtained based on the native surface hydroxyl groups, acting as metal ions chelators. Taking into account this fact, in the present work, the sequential process involving chemical hydrolysis of cellulose and autocatalytic esterification using thioglycolic acid was performed to obtain thiol-functionalized cellulose nanocrystals. The change in chemical compositions was attributed to thiol-functionalized groups and explored via the degree of substitution using a back titration method, X-ray powder diffraction, Fourier-transform infrared spectroscopy and thermogravimetric analysis. Cellulose nanocrystals were spherical in shape and ca. 50 nm in diameter as observed via transmission electron microscopy. The adsorption behavior of such a nanomaterial toward divalent copper ions from an aqueous solution was also assessed via isotherm and kinetic studies, elucidating a chemisorption mechanism (ion exchange, metal chelation and electrostatic force) and processing its operational parameters. In contrast to an inactive configure of unmodified cellulose, the maximum adsorption capacity of thiol-functionalized cellulose nanocrystals toward divalent copper ions from an aqueous solution was 4.244 mg g^−1^ at a pH of 5 and at room temperature.

## 1. Introduction

Heavy metal-contaminated water sources can be derived from uncontrolled manufacturing industries, typically electroplating, metallurgical and mining, etc., which causes harmful impacts on ecological issues and human health during long-term exposure [1,2]. The efficient removal of heavy metals from water has been practically evidenced via mainly adsorption, in which adsorbents are of vital importance to the capacity and the selectivity of such processes [3,4,5]. In comparison with other treatment methods (chemical precipitation, coagulation/flocculation, membrane filtration, ion exchange, etc. [1]), the adsorption approach seems to be better fitted to large-scale water treatment, particularly at low metal concentrations, due to its high efficiency, easy operation and low cost. In general, nanostructured materials have been exploited due to the relationship between their adsorption behavior and their porosity/specific surface area [6,7,8]. Besides the porous structure, more interestingly and challenging, the surface chemistry tuned by functionalized groups has been addressed to the chemical interaction with metal ions, aiming to enhance the metal ions adsorption capacity [9,10]. By this approach, some current achievements have mainly focused on the oxygen-containing (such as hydroxyl, carboxyl or carbonyl) and the nitrogen-containing (such as amino) functional groups. The sorption mechanism can be attributed to ion exchange, coordination or electrostatic interaction [11]. For instance, Huang et al. reported the efficient removal of hexavalent chromium ions, divalent copper ions, divalent lead ions and divalent cadmium ions from wastewater using amino-functionalized graphene oxide grafted by (3-aminopropyl) triethoxysilane, based on amino sorption sites chelated with metal ions and electrostatic interactions between oxygen-containing groups and metal ions [10].

Although the chemical binding between metal ions and functionalized groups has been mostly admitted in such adsorption processes [11], the contributions of physisorption and chemisorption have been uncertainly assessed so far. No doubt, hydrophilic materials possessing porous structures and large surface areas are often the most basic choice for the uptake of metal ions from an aqueous solution [6,7,8]; therefore, the contribution of such a physisorption and chemisorption (probably provoked by the retained functional groups on the surface of sorbents [9,10]) could be unseparated in the framework of many studies. By choosing a suitable sorbent, there is room for a comprehensive evaluation of the role of each adsorption type toward metal ions.

In addition to understanding the adsorption behaviors of carbon [7,8,9,10,11], silica [6,12] and alumina-based nanostructured materials [12,13,14] toward metal ions from an aqueous solution, biomass-based adsorbents have been paid great attention thanks to their available oxygen-containing functional groups (hydroxyl, carbonyl and carboxylic), in addition to their preferences from the ecological economics of sustainability [5]. Among others, cellulose, the most abundant natural polymer on earth, has recently gained more attention for a large spectrum of applications, in particular in the adsorption field for environmental treatment. At a nanoscale, nanocelluloses (involving cellulose nanocrystals, CNCs, with a width of 4–70 nm, a length of 100–6000 nm, a crystallinity index of 54–88%; and cellulose nanofibrils, CNFs, with a width of 20–100 nm, a length of >10,000 nm, lower crystallinity than that of cellulose nanocrystals) possess many predominant features, such as highly thermal and mechanical stability, renewability, biodegradability and non-toxicity [15,16,17]. In fact, the physicochemical features of nanocelluloses can be controlled by their size, morphology and crystallinity, which mostly depend on biomass sources and extraction conditions. The chemical-based production of plant-derived nanocelluloses has mainly relied on a two-step sequential process, involving (i) the removal of the lignin and hemicellulose components, waxes and pectin, etc. to achieve microfibril bundles of cellulose via alkaline and/or bleaching treatments; and (ii) the removal of amorphous regions and breaking of glycosidic bonds between the two anhydroglucose units to achieve nanometer-sized fragments via an acid hydrolysis [18,19,20]. More importantly, the surface modification of nanocelluloses can be efficiently obtained based on the hydroxyl groups, acting as metal ions chelators. Taking into account this fact, well-dispersed metal nanoparticles can be formed on the active sites of nanocelluloses due to the dative bonds between metal ions and oxygen-containing moieties [21,22,23,24]. In particular, thiol functional groups have been evidenced to have a strong affinity with metal surfaces, permitting stabilizing metal nanoparticles on nanocelluloses in which thiol-functionalized nanocelluloses can be performed via silylation [25] or esterification [26,27,28]. Stimulated by advancements in the synthesis of metal nanoparticles, by a similar strategy, thiol functionalization can be introduced to nanocelluloses for metal ion adsorption purposes.

In the strategy to introduce thiol groups using trialkoxysilane reagents, cross-linking and self-condensation can impede the target silylation processes [27]; therefore, current efforts are mainly focused on the direct esterification of various cellulosic sources using organic acids or sulfuric acid as catalysts [26,27,28]. However, the above-mentioned works focused on catalysis in organic synthesis or immobilization of metal nanoparticles. On that account, there is room to shed light on chemical binding with metal ions for the purpose of their uptake. In the present work, the sequential process involving chemical hydrolysis of cellulose and autocatalytic esterification using thioglycolic acid was performed to obtain thiol-functionalized cellulose nanocrystals. The adsorption behavior of such a nanomaterial toward divalent copper ions from an aqueous solution at room temperature was also assessed via isotherm and kinetic studies, also accompanying the processing of its operational parameters and elucidating the adsorption mechanism.

## 2. Materials and Methods

### 2.1. Materials

Unless specifically stated otherwise, all chemicals were utilized as supplied, without any further purification involving cellulose microcrystalline (cotton linters, HiMedia Laboratories, Mumbai, India), sodium hydroxide, hydrochloric acid, sulfuric acid (Xilong Scientific, China), thioglycolic acid (Sigma-Aldrich, Germany), copper(II) standard solution (1000 mg L^−1^ Cu, Sigma-Aldrich, Germany) or 1-(2-Pyridylazo)-2-naphthol (Sigma-Aldrich, Germany).

### 2.2. Fabrication of Thioglycolic Acid-Esterified Cellulose Nanocrystals

The chemical hydrolysis (alkaline pretreatment and acid hydrolysis) of cellulose into nanocrystals was conducted as per our previous reports [23,24], and the functionalization of cellulose nanocrystals was conducted as per previous reports [27,29] with some modifications. In more detail, cellulose (10.0 g) was dispersed in 100.0 mL of 5.0 M NaOH aqueous solution and then heated at 80 °C for 4 h. The solid was filtered and washed with distilled water until reaching neutral pH, air dried overnight, and continuously treated in 100.0 mL of dimethyl sulfoxide at 80 °C for further 4 h. After washing with distilled water, pre-treated cellulose was hydrolyzed at 80 °C for 8 h using 200.0 mL of a mixture containing 1.0 M HCl aqueous solution and 10.0 M H_2_SO_4_ aqueous solution (1/3, *v*/*v*). Cellulose nanocrystals were obtained by centrifuging and washing with distilled water until reaching neutral pH. After freeze drying, cellulose nanocrystals (CNCs, 1.0 g) were esterified using thioglycolic acid (TGA, 0.9 mL, 2 equiv.) in 100.0 mL of hexane at 45–50 °C for 24 h, yielding thioglycolic acid-functionalized cellulose nanocrystals (TGA-CNCs) after centrifuging and washing with acetone and distilled water.

### 2.3. Characterization of Thioglycolic Acid-Esterified Cellulose Nanocrystals

The crystalline structures of cellulose nanocrystals (CNCs) and thioglycolic acid-functionalized cellulose nanocrystals (TGA-CNCs) were examined via X-ray powder diffraction (XRD), measured on a D2 Phaser instrument (Bruker, Germany) with a Cu-Kα radiation (λ = 1.5406 Å), voltage of 40 kV, current of 30 mA and 2θ of 5−65°. Their crystallinity index (%I_C_) values could be estimated according to the XRD peak height method [30,31,32] as Equation (1).

(1) $${\%I}_{C}=\frac{I_{crystalline}-I_{amorphous}}{I_{crystalline}}\times 100$$

where I_crystalline_ and I_amorphous_ represent the maximum and the minimum diffraction intensities for the crystalline and the amorphous phases, respectively.

The functional groups could be discovered based on bands of covalent bond vibrations via Fourier-transform infrared spectroscopy (FT-IR), measured on a Tensor 27 Bruker instrument (Germany) in the wavenumber range of 4000–400 cm^−1^, using the KBr pellet preparation method. The thermal behavior was characterized by thermogravimetric analysis, measured on a Mettler Toledo instrument (Switzerland), under a N_2_ atmosphere with a heating rate of 5 °C/min. The size and the morphology were observed on transmission electron microscopy (TEM), measured on a JEOL JEM 2100F instrument (Japan) at 120 kV, in which TGA-CNCs were dispersed in distilled water and ultrasonicated for 15 min before dropping onto a copper grid for measuring. The particle size distribution was measured on a TEM image with the assistance of ImageJ software.

The degree of substitution (DS) of TGA-CNCs can be determined according to Equation (2) using the back titration method [27]. In principle, TGA-CNCs were reacted with an excess amount of 0.5 M NaOH aqueous solution at 60 °C for 48 h; afterwards, the filtered solution was back-titrated using 0.5 M HCl aqueous solution.

(2) $$DS=\frac{162.14\times C_{M}\times (V_{0}-V)}{1000\times m-C_{M}\times (V_{0}-V)\times ({MW}_{TGA}-{MW}_{H2O})}$$

where: m (g) represents the mass of TGA-CNCs; C_M_ represents the molar concentration of HCl aqueous solution; V_0_ and V (mL) represent the volumes of HCl aqueous solution titrated for the blank sample and the real sample, respectively; MW_TGA_ and MW_H2O_ represent the molar weights of thioglycolic acid and water, respectively; and 162.14 is the molar weight of anhydro glucose unit. The blank sample was titrated in the similar procedure using unfunctionalized CNCs.

The pH zero-point charge (pH_ZPC_) of TGA-CNCs was determined by plotting a curve between the initial pH conditions (2.0–9.0, adjusted by 0.1 M HCl aqueous solution or 0.1 M NaOH aqueous solution) and the differences between the final pH condition and the initial pH condition (ΔpH = pH_f_ − pH_i_), using 0.1 g of sorbent shaken in 100.0 mL of 0.01 M KCl aqueous solution for 24 h equilibration.

### 2.4. The Uptake of Divalent Copper Ions on Thioglycolic Acid-Esterified Cellulose Nanocrystals

Typically, thioglycolic acid-esterified cellulose nanocrystals (TGA-CNCs) were added to an aqueous solution of divalent copper ions at 5 ppm (an adsorbent concentration of 1.0 mg mL^−1^), adjusted to a pH of 5 and stirred at room temperature for up to 30 min. The effects of processing parameters on adsorption efficiency were examined, involving the contact time (up to 30 min) and the initial divalent copper ion concentration (3 ppm, 5 ppm and 10 ppm). At a time interval, an aliquot portion was taken out, and the copper ion concentration was quantified by ultraviolet-visible spectrophotometry (UV-vis, Libra S22) using 1-(2-Pyridylazo)-2-naphthol (PAN) reagent and measured at a wavelength of 545 nm.

The uptake of divalent copper ions from an aqueous solution of thioglycolic acid-esterified cellulose nanocrystals (TGA-CNCs) was evaluated via the adsorption capacity (q; mg g^−1^) according to Equation (3).

(3) $$q=\frac{(C_{0}-C_{t})\times V}{1000\times m}$$

where: C_0_ and C_t_ (ppm) = the divalent copper ions concentrations at the initial and the time interval, respectively; V (mL) = the solution volume; and m (g) = the weight of adsorbent.

Accordingly, isotherm and kinetic studies were conducted under the following conditions: typically, TGA-CNC nanomaterial (1.0 mg mL^−1^) was added to an aqueous solution of divalent copper ions (3 ppm, 5 ppm and 10 ppm) at a pH of 5 and stirred for up to 30 min at room temperature.

## 3. Results and Discussion

### 3.1. Characteristics of Thioglycolic Acid-Esterified Cellulose Nanocrystals

**Scheme 1:**
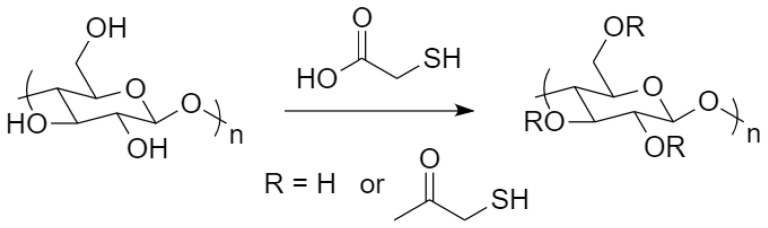
Autocatalytic esterification of cellulose nanocrystals (CNCs) and thioglycolic acid (TGA) to yield thioglycolic acid-esterified cellulose nanocrystals (TGA-CNCs).

In general, the esterification of nanocellulose was often carried out using an organic catalyst, typically ʟ-tartaric acid, citric acid, acetic acid [27], *p*-toluenesulfonic acid [28] or lipase [29]. In the present work, the autocatalytic esterification of cellulose nanocrystals and thioglycolic acid was proceeded with the help of thioglycolic acid as an organocatalyst. In fact, the strength of thioglycolic acid (pK_a_ = 3.83) is equivalent to previously reported organic acids, such as ʟ-tartaric acid (pK_a1_ = 2.89), citric acid (pK_a1_ = 3.13), and acetic acid (pK_a_ = 4.76). The esterification process to form the thiol-functionalized groups (CNCs-SH) was obtained via the ester bonds (Scheme 1) and its efficiency was estimated via the degree of substitution (DS) (Equation (2)). The obtained degree of substitution of 0.12 is in the similar range as the thioglycolic acid-esterification catalyzed by the above-mentioned organic acids [27]; with a relatively low value of degree of substitution, only surface hydroxyl groups on nanocellulose were successfully esterified.

**Figure 1:**
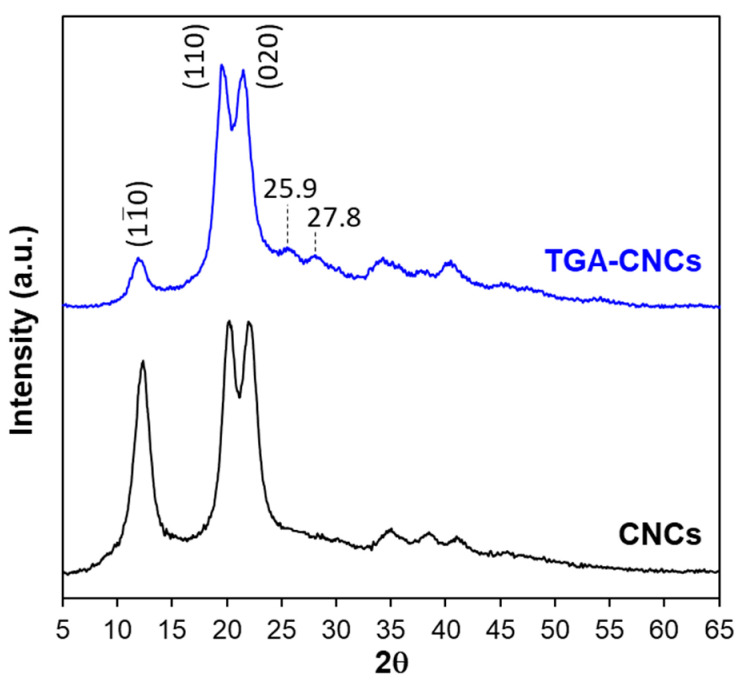
XRD patterns of cellulose nanocrystals (CNCs) and thioglycolic acid-esterified cellulose nanocrystals (TGA-CNCs).

The crystalline structures of cellulose nanocrystals (CNCs) and thioglycolic acid-esterified cellulose nanocrystals (TGA-CNCs) were examined by powder XRD patterns (Figure 1), assigning to cellulose type II with the diffraction peaks at 2θ of 12.0°, 20.0° and 22.0° associated to the (1$\bar{1}$0), (110) and (020) planes, respectively [30]. According to the XRD peak height method (Equation (1)) [31,32], the crystallinity index of TGA-CNCs (84.6%) was equivalent to that of CNCs (82.4%). Celluloses possess both crystalline and amorphous regions, and they can be presented in various polymorphs in which the hydrogen bonding network determines their molecular arrangement, reflecting the typical polymorph. In acid hydrolysis, a strong acid attacks the amorphous regions; meanwhile, a swell of the crystalline regions occurs, leading to their rearrangement; therefore, the polymorph can be changed [30]. In this work, in the stage of esterification using thioglycolic acid, the polymorphic transformation was not obtained in which the transformation from cellulose type I (characterized by the diffraction peaks at 2θ of 14.5°, 16.5° and 22.5° associated to the (1$\bar{1}$0), (110) and (200) planes) to cellulose type II has been universally admitted to be irreversible [30]. In short, any changes observed in the XRD patterns between TGA-CNCs and CNCs all pointed out the surface thiol-functionalization of CNCs with thioglycolic acid; typically, the intense peak at 2θ of 12.0° and the additional peaks at 2θ of 25.9° and 27.8°.

**Figure 2:**
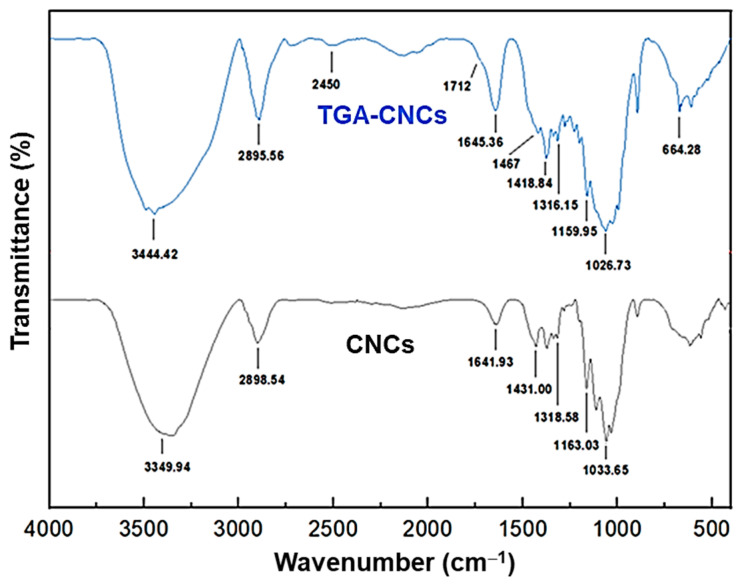
FT-IR spectra of cellulose nanocrystals (CNCs) and thioglycolic acid-esterified cellulose nanocrystals (TGA-CNCs).

The chemical compositions of cellulose nanocrystals (CNCs) and thioglycolic acid-esterified cellulose nanocrystals (TGA-CNCs) were explored via FT-IR spectroscopy (Figure 2). The absorption bands characterized to cellulose were presented, such as 3444.4 cm^−1^ (O–H stretching), 2895.5 cm^−1^ (C–H stretching), 1645.3 cm^−1^ (O–H bending from absorbed H_2_O), 1316.1 cm^−1^ (CH_2_ rocking at the C6), 1159.9 cm^−1^ (C–O–C stretching), 1026.7 cm^−1^ (C–OH stretching), 664.2 cm^−1^ (C–OH out-of-plane bending), etc. [23,24,27,29]. In the sample of TGA-CNCs, the additional vibrational bands at 2450 cm^−1^ (S–H stretching), 1712 cm^−1^ (COO– asymmetric stretching) and 1467 cm^−1^ (COO– symmetric stretching) evidenced the presence of thiol and carboxylate groups, in spite of the weak intensities [27,28,29]. These results confirmed the successful autocatalytic esterification of CNCs with TGA, as also proven via the above degree of substitution.

**Figure 3:**
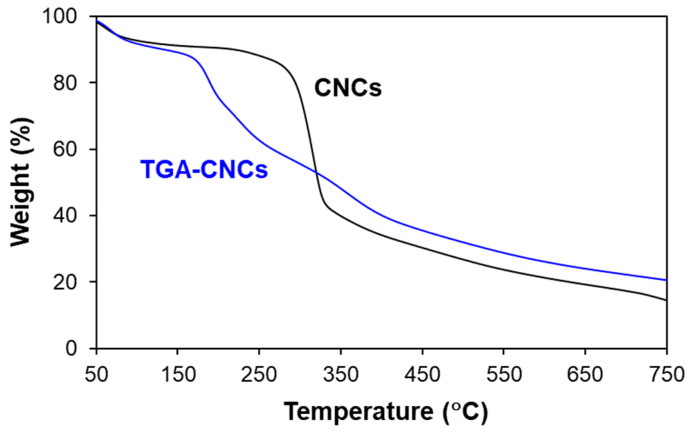
Thermogravimetric analysis of cellulose nanocrystals (CNCs) and thioglycolic acid-esterified cellulose nanocrystals (TGA-CNCs).

The differences in thermogravimetric behavior between cellulose nanocrystals (CNCs) and thioglycolic acid-esterified cellulose nanocrystals (TGA-CNCs) were attributed to thiol functionalization on the surface of nanocellulose (Figure 3). In fact, the mass loss of absorbed water (around 8 wt%) was observed on both the samples at 100 °C; meanwhile, the thermal decomposition of CNCs started at around 275 °C, being higher than that of TGA-CNCs (started at around 175 °C). The weight losses of CNCs were 45.8% (275–350 °C) and 25.4% (350–750 °C), and the weight losses of TGA-CNCs were 27.3% (175–275 °C), 10.6% (275–350 °C) and 27.6% (350–750 °C). It is noted that the thermal stability of cellulose was previously evidenced around 250 °C [23]; therefore, the mass loss of TGA-CNCs from around 175 °C was contributed to by an amount of thiol groups on the surface. Furthermore, such thermal decomposition of TGA-CNCs seemed to be two sequential steps (starting at around 175 °C and around 350 °C). During the second step, the decomposition temperature of TGA-CNCs was slightly higher than that of CNCs, which can be ascribed to the presence of carboxylate groups. In this sense, the material surface could be coated with a caramelization effect, accelerated by carboxylate groups; as a consequence, the thermal decomposition of TGA-CNCs was slower than that of CNCs [33,34].

**Figure 4:**
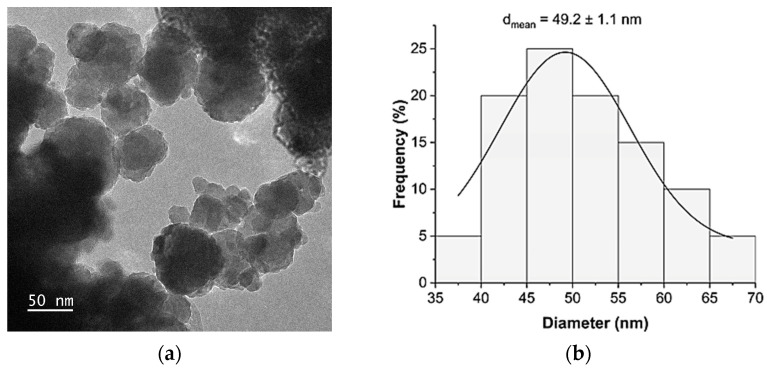
(**a**) TEM image of thioglycolic acid-esterified cellulose nanocrystals (TGA-CNCs); (**b**) their particle size distribution histogram.

In addition, the size and the morphology of thioglycolic acid-esterified cellulose nanocrystals (TGA-CNCs) were observed on a TEM image, indicating their spherical shape (Figure 4a), and their particle size distribution showed a mean diameter of ca. 50 nm (Figure 4b). However, the agglomeration of small spheres could result in larger sizes due to the supramolecular network of cellulose nanocrystals [23,35]. In nature, cellulose possesses both highly stable crystalline and disordered amorphous regions. The most accessible glycosidic bonds can be broken by an acid reagent; as a consequence, cellulose was subdivided into nanosized fragments as nanospheres in this work.

### 3.2. Processing Parameters for the Uptake of Divalent Copper Ions on Thioglycolic Acid-Esterified Cellulose Nanocrystals: Isotherm and Kinetic Studies

**Figure 5:**
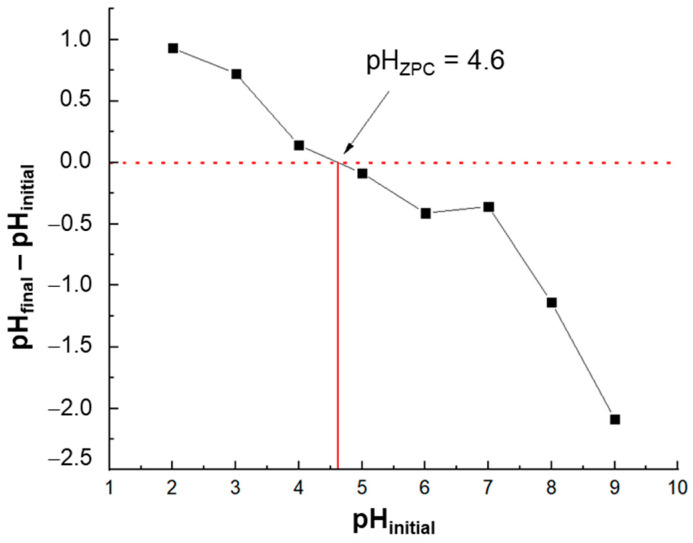
The pH zero-point charge of thioglycolic acid-esterified cellulose nanocrystals (TGA-CNCs).

In principle, the uptake of divalent copper ions from an aqueous solution on thioglycolic acid-esterified cellulose nanocrystals (TGA-CNCs) was expected to have relied on the electrostatic interaction between oppositely charged counterparts [36]; therefore, its adsorption efficiency was dependent on the initial pH condition. In the present work, the optimal condition should be at a pH of 4–5, in agreement with the pH zero-point charge (pH_ZPC_) of 4.6 (Figure 5). The rationale behind this fact is due to the equivalence of positive and negative charges on the adsorbent surface. At a pH < pH_ZPC_, the surface was protonated, leading to the electrostatic repulsion with copper cations and thus lowering metal ion adsorption. At a pH > pH_ZPC_, the adsorbent surface gained more negative charges, strengthening the electrostatic forces with positively charged ions; however, at higher pH conditions (pH > 6), the formation of copper hydroxide precipitates could prevent the uptake of copper ions [37]. In this context, an excess amount of H_3_O^+^ or OH^−^ can block the adsorptive sites, interfering with metal ion adsorption. In short, the pH condition of 5 could be optimal for the uptake of divalent copper ions from an aqueous solution on thioglycolic acid-esterified cellulose nanocrystals.

**Figure 6:**
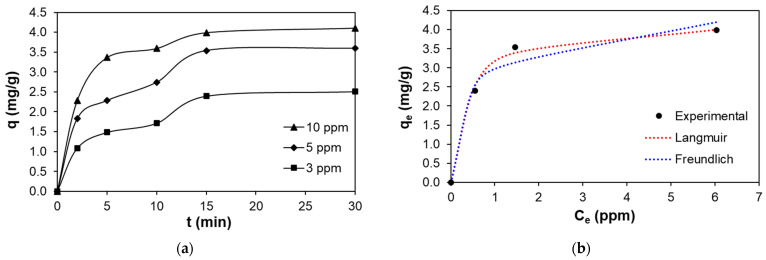
(**a**) The dependence of copper ion adsorption capacity of TGA-CNCs on contact time and initial copper ion concentration; (**b**) Copper adsorption isotherm study of TGA-CNCs at a pH of 5 and room temperature.

This section aims to explore the equilibrium for the uptake of divalent copper ions from an aqueous solution on thioglycolic acid-esterified cellulose nanocrystals (TGA-CNCs) via its adsorption capacity, varied on the contact time and the initial concentration (Figure 6a). In the range of initial copper ion concentrations of 3–10 ppm, all the adsorption processes reached the equilibrium after a contact time of 15 min at a pH of 5 and room temperature, using 1.0 mg mL^−1^ adsorbent. At an initial copper ion concentration of 3 ppm, the equilibrium adsorption capacity was much lower, due to the preponderance of the number of adsorptive sites on TGA-CNCs over the number of copper ions in an aqueous solution; thus, the copper adsorption capacity of TGA-CNCs dramatically increased with a rise in initial copper ion concentrations from 3 ppm to 5 ppm. However, such adsorptive sites could be agglomerated at higher copper ion concentrations, resulting in the competitive anchors on the surface of TGA-CNCs and thus a slight increase in adsorption capacity from 5 ppm to 10 ppm [7]. For all initial copper ion concentrations, a steeper gradient was observed in the first 5 min interval, indicating a faster adsorption rate due to a large amount of available adsorptive sites. In the next intervals of 5 min and 10 min, the lower slopes evidenced a decrease in adsorption rate, relating to the adsorptive sites partially filling up with adsorbed copper ions [7].

It is worth mentioning that an insignificant amount of copper ions adsorbed on unmodified cellulose (less than 0.2 mg g^−1^) was found at an initial copper ion concentration of 5 ppm for a contact time of 30 min at a pH of 5 and room temperature. This result evidenced an inactive configuration of the natural surface hydroxyl groups of CNCs toward the uptake of metal ions. In this sense, the thiol groups better boosted the metal chelation, and thus the uptake of metal ions seemed to be mainly governed by chemical interactions (probably involving ion exchange, metal chelation or electrostatic force) [9,10,11].

Subsequently, the experimental data for the adsorption of divalent copper ions from an aqueous solution on thioglycolic acid-esterified cellulose nanocrystals (TGA-CNCs) were predicted according to the Langmuir and the Freundlich isotherm models (Figure 6b) [6,7,10,38,39]. Their corresponding isotherm parameters were also given in Table 1. In comparison with a lower coefficient of determination (R^2^ = 0.8483) from the Freundlich isotherm model, such an adsorption seemed to be better fitted with the Langmuir isotherm model (R^2^ = 0.9995) with the maximum adsorption capacity of 4.244 mg g^−1^. In addition, the value of R_L_ (0 < R_L_ < 1) also evidenced a favorable adsorption with a monolayer sorption consisting of copper ions anchored on localized sites of TGA-CNCs. The absorbed molecular interactions did not exist, resulting in homogeneous surface energy and adsorption sites due to the chemical forces between positively charged copper ions and the negatively charged surface of TGA-CNCs. On the contrary, the Freundlich isotherm model describes a multilayer adsorption on a heterogeneous surface, which was not well fitted with the experimental data.

**Figure 7:**
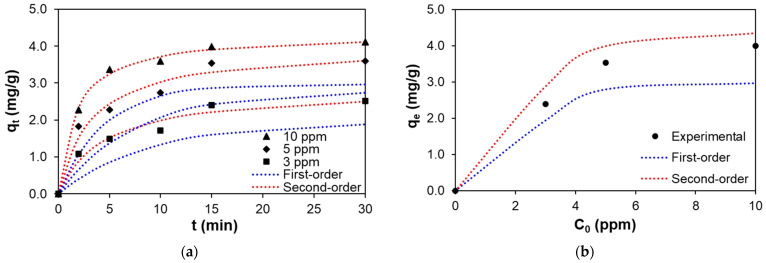
The uptake of divalent copper ions on TGA-CNCs simulated by pseudo-first order (PFO) and pseudo-second order (PSO) kinetics at a pH of 5 and room temperature: (**a**) adsorbed copper ions at a time interval; (**b**) adsorbed copper ions at the equilibrium.

The uptake of divalent copper ions from an aqueous solution on thioglycolic acid-esterified cellulose nanocrystals (TGA-CNCs) was also simulated according to the linear pseudo-first order (PFO) and the linear pseudo-second order (PSO) kinetic models [7,10,39]. Their corresponding kinetic parameters were also given in Table 2. The linear PFO model showed a lower coefficient of determination (R^2^ < 0.90); meanwhile, the experimental results showed a better fit for the linear PSO model (R^2^ > 0.98). In fact, the amounts of copper ions adsorbed on TGA-CNCs at any time interval (Figure 7a) and the equilibrium (Figure 7b) could be estimated from the linear PSO model, which were in the vicinity of the experimental data. In short, with a better linearity and closer predicted level of adsorbed copper ions to the experimental data, the uptake of divalent copper ions from an aqueous solution on the surface of TGA-CNCs obeyed the linear PSO kinetic model, which was ascribed to chemical interactions instead of physical exchange. In addition, the results also confirmed adsorption as a rate-controlling mechanism rather than a diffusion mechanism.

Taking into account the recognition of the Langmuir isotherm model and the linear pseudo-second order kinetic model, the chemisorption mechanism can be involved in the uptake of divalent copper ions from an aqueous solution on the surface of thioglycolic acid-esterified cellulose nanocrystals (TGA-CNCs) via three different mechanisms (ion exchange, metal chelation and electrostatic force), as follows [10].

(i) The ion exchange between copper ions and protons from –OH or –SH groups;

–OH + Cu^2+^ → –OCu^+^ + H^+^

2 –OH + Cu^2+^ → –OCuO– + 2 H^+^

–SH + Cu^2+^ → –SCu^+^ + H^+^

2 –SH + Cu^2+^ → –SCuS–+ 2 H^+^

(ii) The surface metal chelation between copper ions and thiol groups;

2 –SH + Cu^2+^ → (–SH)_2_Cu^2+^

(iii) The electrostatic forces between positively charged copper ions and negatively charged groups (hydroxyl, carbonyl, thiolate).

To sum up, under a static condition, the batch procedure could be established by blending an adsorbent in a wastewater solution containing heavy metal ions (with an appropriate adsorbent concentration) at a pH of 5 and stirring for up to 30 min at room temperature. The adsorption efficiency was affected by not only the adsorbent dosage but also the features of selected metal ions.

In this work, TGA-CNCs were dispersed in an aqueous solution for batch adsorption experiments. The separation of adsorbent can be obtained by centrifugation due to its supramolecular network and the desorption of metal ions using a strong acid solution (like HCl, HNO_3_, etc.). The recycling efficiency should be evaluated via its cumulative adsorption capacity within several cycles. In order to improve the removal of metal ions and the recyclability, progressive nanocelluloses are currently underway involving nanocellulose-based aerogel or membranes.

## 4. Conclusions

Taking into account the metal chelation of functionalized groups on cellulose nanocrystals surface, autocatalytic thioglycolic acid esterification occurred toward the uptake of divalent copper ions from an aqueous solution. Cellulose nanocrystals were spherical in shape and ca. 50 nm in diameter, with a degree of thiol substitution of 0.12. The chemical structure of thiol-functionalized cellulose nanocrystals was beneficial to chemisorption of positively charged copper ions via three different mechanisms involving (i) ion exchange between copper ions and protons from -OH or -SH groups; (ii) surface metal chelation between copper ions and thiol groups; and (iii) electrostatic forces between positively charged copper ions and negatively charged groups (hydroxyl, carbonyl, thiolate). Consistent with the chemisorption mechanism, the experimental data were better interpreted according to the Langmuir isotherm model and the linear pseudo-second order kinetic model, permitting the anticipation and processing of operational parameters for the uptake of divalent copper ions on such a nanomaterial.

## Figures and Tables

**Table 1:** Isotherm parameters for the uptake of divalent copper ions on TGA-CNCs at a pH of 5 and room temperature.

Isotherm Models		Parameters		R^2^
Langmuir	$\frac{C_{e}}{q_{e}}=\frac{1}{K_{L}q_{m}}+\frac{C_{e}}{q_{m}}$ $R_{L}=\frac{1}{1+K_{L}C_{0}}$	q_m_ (mg g^−1^)	4.244	0.9995
		K_L_ (L mg^−1^)	2.733	
		R_L_	0.035–0.109	
Freundlich	$lnq_{e}=lnK_{F}+\frac{1}{n}lnC_{e}$	n	4.885	0.8483
		K_F_ (mg g^−1^)(L mg^−1^)^1/n^	2.903	

Where: C_0_ and C_e_ = the copper ions concentrations at the initial and the equilibrium, respectively; q_e_ and q_m_ = the equilibrium and the maximum adsorption capacities, respectively; K_L_ and R_L_ = the Langmuir and the dimensionless equilibrium constants, respectively; K_F_ and n = the Freundlich and the heterogeneity constants, respectively; R^2^ = the coefficient of determination.

**Table 2:** Kinetic parameters for the uptake of divalent copper ions on TGA-CNCs at pH of 5 and room temperature.

Kinetic Models		Parameters	Initial Copper Concentrations		
			3 ppm	5 ppm	10 ppm
Pseudo-first order	$\ln\left(q_{e}-q_{t}\right)=lnq_{e}-kt$	q_e_ (mg g^−1^)	1.937	2.794	2.967
		k (min^−1^)	0.1158	0.1357	0.2242
		R^2^	0.8696	0.8899	0.8930
Pseudo-second order	$\frac{t}{q_{t}}=\frac{1}{kq_{e}^{2}}+\frac{t}{q_{e}}$	q_e_ (mg g^−1^)	2.880	3.992	4.352
		k (g mg^−1^ min^−1^)	0.0778	0.0786	0.1352
		R^2^	0.9817	0.9908	0.9992

Where: q_e_ and q_t_ = the adsorbed copper ions at the equilibrium and a time interval (min), respectively; k = the rate constant; R^2^ = the coefficient of determination.

## Data Availability

The data presented in this study are available within the manuscript.
